# Imaging extrachromosomal DNA (ecDNA) in cancer

**DOI:** 10.1007/s00418-024-02280-2

**Published:** 2024-04-16

**Authors:** Karin Purshouse, Steven M. Pollard, Wendy A. Bickmore

**Affiliations:** 1 MRC Human Genetics Unit, Institute of Genetics and Cancer, University of Edinburgh, Edinburgh, UK; 2 Centre for Regenerative Medicine, Institute for Regeneration and Repair & Cancer Research UK Scotland Centre, University of Edinburgh, Edinburgh, UK; 3 Edinburgh Cancer Research UK Centre, University of Edinburgh, Edinburgh, UK

**Keywords:** Oncogene, Double-minute, Fluorescence in situ hybridisation, Homogeneously staining regions, Transcription hubs

## Abstract

Extrachromosomal DNA (ecDNA) are circular regions of DNA that are found in many cancers. They are an important means of oncogene amplification, and correlate with treatment resistance and poor prognosis. Consequently, there is great interest in exploring and targeting ecDNA vulnerabilities as potential new therapeutic targets for cancer treatment. However, the biological significance of ecDNA and their associated regulatory control remains unclear. Light microscopy has been a central tool in the identification and characterisation of ecDNA. In this review we describe the different cellular models available to study ecDNA, and the imaging tools used to characterise ecDNA and their regulation. The insights gained from quantitative imaging are discussed in comparison with genome sequencing and computational approaches. We suggest that there is a crucial need for ongoing innovation using imaging if we are to achieve a full understanding of the dynamic regulation and organisation of ecDNA and their role in tumourigenesis.

## Introduction

Extrachromosomal DNAs (ecDNA) were identified almost 60 years ago, but the last decade has seen renewed interest in their roles in cancer and oncogene amplification. EcDNA are often the location of all key oncogene amplifications and facilitate intra-tumoural copy number heterogeneity, as well as being associated with treatment resistance and poor prognosis in cancer (Turner et al. 2017; Kim et al. 2020). Recent evidence suggests that ecDNA evolve during cancer progression (Luebeck et al. 2023).

EcDNA were first discovered in tumours using light microscopy (Cox et al. 1965; Lubs and Salmon 1965) and have gone on to be studied by both imaging and genomic tools. Key questions of ecDNA behaviour have mainly been explored through DNA sequencing and computational approaches. However, imaging remains vital to truly understand ecDNA dynamics in and between cells, with many questions remaining around gene expression, regulation and spatial organisation. As part of this Special Issue ‘Visualizing genomes: the centennial of the Feulgen reaction’ this review highlights the important role played by being able to image DNA in furthering the understanding of ecDNA organisation and regulation, and we discuss the important opportunities for innovation.

## An overview of ecDNA structure

**Fig. 1:**
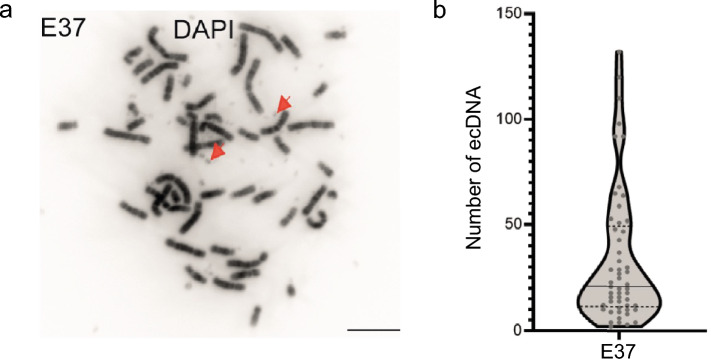
ecDNA at metaphase. **A** 4′,6-Diamidino-2-phenylindole (DAPI) stained metaphase spreads from a recurrent glioblastoma cell line E37. ecDNA appear as small DAPI-stained dots (arrowed). Scale bar: 10 µm. **B** Violin plot of number of ecDNA per metaphase spread in E37 cells, median and quartiles are shown. Number of metaphase spreads = 53

EcDNA were first described when karyotype analyses of human cancers revealed abnormal chromosomal, and additional non-chromosomal, structures in metaphase spreads. Tumours resected from patients were found to harbour multiple small DNA fragments characterised as centromere-free double chromatin bodies (later defined as double minutes) and very long, abnormal chromosomes later defined as homogeneously staining regions (HSRs) (Lubs and Salmon 1965; Cox et al. 1965; Biedler and Spengler 1976). The existence of these chromatin bodies as doublets or singlets has resulted in the more global description of these chromosome-independent bodies as ecDNA (Hamkalo et al. 1985) (Fig. 1).

Their varied genomic composition has led to the proposal that multiple mechanisms may contribute to ecDNA formation (Wang et al. 2021). These include breakage-fusion-bridge cycles (McClintock 1938), chromothripsis (Ly and Cleveland 2017; Stephens et al. 2011; Rosswog et al. 2021; Shoshani et al. 2021), translocation-(excision)-deletion-amplification (Röijer et al. 2002; Van Roy et al. 2006) and episome formation (Carroll et al. 1988; Vogt et al. 2004; Storlazzi et al. 2010). Available evidence suggests that a sizeable proportion of ecDNA derive their origins from chromothripsis; however, the structure of ecDNA indicate cases where other, or indeed multiple, models can explain their origin. It remains unclear what triggers such genome rearrangement events.

EcDNA often exist as singlets, with only 30% shown to be paired doublets (Turner et al. 2017). EcDNA can re-integrate into chromosomes to form more stable chromosomal focal amplifications – HSRs (Balaban-Malenbaum and Gilbert 1980; Hamkalo et al. 1985; Vogt et al. 2004; Storlazzi et al. 2010; Verhaak et al. 2019). Genomic sequencing and associated analytical tools have enabled higher resolution structural characterisation of ecDNA. EcDNA in cancer cells had originally been hypothesised to be circular on the basis of comparison with similar structures in other organisms such as protozoa (Schimke 1984). Combining sequencing tools with microscopy has confirmed that ecDNA are indeed circular and generally approximately 1–3 Mb in size, although this may extend up to 5 Mb (Turner et al. 2017; Deshpande et al. 2019; Verhaak et al. 2019; Wu et al. 2019). Their large size differentiates ecDNA from other circular extrachromosomal structures, such as extrachromosomal circular DNA (eccDNA), which encompasses various types with differing characteristics and functions (Wang et al. 2021) (Table 1).

**Table 1:** Characteristics of various extrachromosomal circular DNA structures

Structure	Size	Characteristics	References
EcDNA	1–5 Mb	Singlets or doublets (formerly described as double minutes), harbour genes and regulatory elements. Rare in non-cancer cells	Turner et al. 2017 Deshpande et al. 2019 Verhaak et al. 2019 Wu et al. 2019
EccDNA	0.1 Kb–1 Mb	Rarely harbour genes/regulatory elements unless size allows, seen in normal and cancer cells	Møller et al. 2018 Wang et al. 2021
Telomeric circles (or C-circles)	100 bp to 30 Kb – integral multiples of 738 bp	Provide a specialised mechanism for telomere elongation via the alternative mechanism of telomere (ALT) mechanism, multiples of 738 bp	Reddel 2003 Henson et al. 2009 Basenko et al. 2010
microDNA	100–400 bp	Seen in normal mouse and human cell lines, involved in small regulatory RNAs, such as miRNA generation	Shibata et al. 2012 Paulsen et al. 2018 Noer et al. 2022
Small polydispersed circular DNA (spcDNA)	100 bp–10 Kb	Earliest description of eccDNA as described in HeLa cells, linked to genomic instability	Smith and Vinograd 1972 Regev et al. 1998

EcDNA and HSRs were confirmed as the location of oncogene amplifications in a range of glioma, neuroblastoma and colorectal cell lines (Alitalo et al. 1983; Kohl et al. 1983; Bigner et al. 1987). Across many cancers, the most common focal oncogene amplifications have been shown to be all located on ecDNA and/or HSRs. This enables oncogene copy number to be amplified tens to hundreds of times, with significant intra- and inter-tumoral copy number heterogeneity (Turner et al. 2017; Lange et al. 2022). Given that ecDNA replicate only once per cell cycle (Barker et al. 1980), it has been suggested that oncogene amplification occurs via random segregation at mitosis with subsequent cell selection favouring ecDNA-harbouring cells (Lange et al. 2022).

In addition to their resident oncogenes, ecDNA also harbour regulatory elements (enhancers) required to drive oncogene expression (Morton et al. 2019). EcDNA-resident enhancers have been proposed to interact with oncogenes in cis and trans (Helmsauer et al. 2020; Zhu et al. 2021). It has also been suggested that ecDNA harbour regulatory elements that are independent of their relevant oncogenes and that facilitate trans-activation between enhancers and other ecDNA-inhabiting oncogenes (Hung et al. 2022). This has led to debate around transcription regulation in the context of ecDNA. While some studies suggest that circular amplicons result in augmented copy-number normalised transcription in comparison with non-circular amplicons, other studies indicate a simple linear relationship between ecDNA copy number and gene expression with levels of transcription per ecDNA no different from that of the endogenous chromosomal loci (Wu et al. 2019; Kim et al. 2020; Purshouse et al. 2022; Stöber et al. 2023). Recent data indicate high intercellular and intranuclear heterogeneity of ecDNA transcription, suggesting that ecDNA transcriptional dynamics are highly complex (Chamorro González et al. 2023; Stöber et al. 2023).

## EcDNA and cancer

EcDNA are a frequent feature of many cancer types but are very rare in normal tissue (Benner et al. 1991; Turner et al. 2017; Kim et al. 2020). Although analysis of the Mitelman database initially suggested ecDNA were present in only 1.4% of cancers (Fan et al. 2011), an integrated study combining whole genome sequencing (WGS) and imaging, using primarily cancer cell lines across 17 cancer types, identified ecDNA in nearly half of cancers (Turner et al. 2017). A subsequent WGS study from 3212 patients with cancer and 1810 non-cancer samples showed 14.3% of tumour samples harboured ecDNA and in 25 of 29 cancer types (Kim et al. 2020). EcDNA are particularly common in glioblastoma, with large-scale analysis of WGS data showing that ~ 50–60% of glioblastoma cells carry ecDNA, rising to 90% in patient-derived glioblastoma tumour models (Turner et al. 2017; Kim et al. 2020). Other cancers with high ecDNA occurrence include sarcoma and oesophageal cancers (Kim et al. 2020). Linking clinical and WGS data has shown that ecDNA amplification is associated with worse 5-year survival outcomes, although ecDNA level was not associated with metastatic status or previous cancer treatment (Turner et al. 2017; Kim et al. 2020). Importantly, ecDNA have been exposed as an early event in cancer, having been identified in dysplastic cells prior to the development of oesophageal adenocarcinoma, with ecDNA copy number and structure evolving during cancer progression (Luebeck et al. 2023).

There are dynamic ecDNA responses to cancer treatments. In a range of *ecMYC* (*c*-*MYC*) cancer cell lines treatment with hydroxyurea, which inhibits ribonucleotide reductase, resulted in a marked reduction in *c*-*MYC* copy number that was not observed in a cell line harbouring *c*-*MYC* on an HSR (Colo320HSR) (Von Hoff et al. 1992). Hydroxyurea also reduces copy number of ecDNA-amplified oncogenes in a range of in vitro tumour contexts (Eckhardt et al. 1994; Canute et al. 1998; Shimizu et al. 2007). EcDNA dynamics may play a role in targeted treatment resistance. GBM39 glioblastoma cells with high levels of mutant *EGFRvIII*-containing ecDNA have increased receptor tyrosine kinase signalling and cell proliferation, and show reduced apoptosis and enhanced cell death in the presence of the EGFR inhibitor erlotinib. Erlotinib resistance was accompanied by a significant reduction in *EGFRvIII* ecDNA, but maintenance of *EGFR* HSRs, likely to be a response to evade drug-induced cell death (Nathanson et al. 2014; Turner et al. 2017). In *BRAF*-mutant ecDNA-null melanoma cells, *BRAF* amplification – primarily via ecDNA formation – developed following Raf/MEK-inhibitor treatment. A preference for ecDNA to HSR conversion was observed during stable dual drug dosing, but rare ecDNA could also re-emerge from a predominantly HSR-*BRAF* population (Song et al. 2021).

Radiotherapy, a treatment modality that results in DNA strand breaks, also leads to ecDNA evolution. Epidermoid cells with ecDNA harbouring the drug resistance gene *Multidrug Resistance 1* (*MDR1*) and Colo320 cells harbouring ec*MYC* were both shown to lose ecDNA copy number following ionising radiation, with *ecMDR1* relocated to micronuclei (Sanchez et al. 1998; Schoenlein et al. 2003). In contrast, a study in established cell lines harbouring ecDNA (Colo320), or driven to form ecDNA by methotrexate (MTX) resistance, showed that both random DNA damage caused by ionising radiation or doxorubicin, and targeted nuclease-induced DNA damage near the amplified ecDNA gene *DHFR*, drove ecDNA to form ectopic chromosome integrations (Shoshani et al. 2021).

These studies highlight that ecDNA are an important feature of cancer that are affected by anti-cancer treatment by mechanisms yet to be fully understood. It remains unclear whether ecDNA represent an important therapeutic target or are merely a downstream consequence of upstream cellular events, given that cancers can occur and be aggressive in the absence of ecDNA.

## Experimental models for the study of ecDNA

Before considering the importance of direct visualisation by microscopy, we first describe the different cellular models that can be used for the study of ecDNA. These can be broadly considered in three categories: established cell lines, drug-based selection and primary patient samples.

### Established cell lines

EcDNA are often studied in a limited repertoire of established cell lines. For example, the Colo320DM and Colo320HSR cell lines were derived from a patient with cancer of the sigmoid colon, and were among the first cells where oncogene amplification, of *c*-*MYC,* was localised to DM or HSR sites, respectively (Quinn et al. 1979; Alitalo et al. 1983). These ecDNA have more recently been characterised as 4.33 Mb in size, each carrying multiple copies of *c*-*MYC* (Hung et al. 2021). Histopathology described moderately undifferentiated adenocarcinoma, atypical pseudo-glands with an area of poorly differentiated carcinoid. Hormone and polypeptide levels were atypical for colorectal cancer, e.g. low carcinoembryonic antigen (CEA), and more characteristic of a neuroendocrine carcinoma. This suggests that Colo320 may be highly atypical of colorectal carcinoma cell lines (Quinn et al. 1979). Other examples of extensively-studied established cell lines with resident ecDNA include PC3 (prostate cancer – *c*-*MYC* ecDNA), SNU16 [gastric cancer – *c*-*MYC* and *fibroblast growth factor receptor 2* (*FGFR2*) ecDNA] and TR14 (neuroblastoma – *c*-*MYC* ecDNA) (Kaighn et al. 1979; Cowell and Rupniak 1983; Park et al. 1990; Hung et al. 2021).

It is unclear how well-established ecDNA cell lines, many over 40 years old, accurately represent tumour biology. The selection pressure for survival in culture over extended periods of time may have enriched for cells with atypical features. While the presence of ecDNA in mouse models of cancer is not well documented, a Cre-recombinase strategy has recently been used to engineer ecDNA into cell lines and mice that normally do not harbour them (Pradella et al. 2023). This has the potential to advance our understanding of the role of ecDNA in the early stages of tumour initiation and to follow their dynamics in vivo.

### Drug-based cell selection

Selection methods have been used to study the origins and evolution of ecDNA (Alt et al. 1978; Kaufman et al. 1979). Low-dose, continuous, MTX drives the formation of *DHFR*-harbouring ecDNA and HSRs in HeLa (cervical) and H29T (colorectal) cancer cell lines (Shoshani et al. 2021). A similar strategy using a BRAF/MEK inhibitor combination resulted in *BRAF*-harbouring ecDNA forming in a previously ecDNA-null melanoma cell line (Song et al. 2021). Many such studies include the development of single cell clones and are reliant on continuous drug exposure. This may not be directly relevant to the heterogeneity observed in cancers, where ecDNA exist de novo rather than in response to drug-based selection.

### Primary patient samples

EcDNA were originally discovered in patient samples. EcDNA are markedly less frequent in established cell lines in comparison with primary human tumours, with the former also harbouring a higher proportion of HSRs (Benner et al. 1991). This was corroborated in a study that identified ecDNA in ~ 40% of tumour cell lines and ~ 90% of patient-derived brain tumour models (Turner et al. 2017). Examples of patient-derived models include GBM39, an *ecEGFRvIII-*expressing xenograft cell line comprising cells originally resected from a patient with primary glioblastoma (Sarkaria et al. 2007; Nathanson et al. 2014). The HK359 glioblastoma cell line, which also harbours *ecEGFRvIII*, was derived directly into neurosphere culture from a heavily pre-treated patient with recurrent glioblastoma (Laks et al. 2016). Other examples of primary cells used in recent studies include primary neuroblastoma cells (Helmsauer et al. 2020; Hung et al. 2021; Lange et al. 2022; Stöber et al. 2023) and primary glioblastoma cell culture (deCarvalho et al. 2018; Purshouse et al. 2022) (Fig. 1A). Genomic profiles indicate good molecular synergy between the tumours, their derived cell cultures, and subsequent xenografts (deCarvalho et al. 2018).

Patient-derived cell cultures likely represent the best tool for studying established ecDNA and recapitulating the complexities of cancer and ecDNA biology. However, the inherent lack of a ‘normal’ pre-pathology comparator cell culture generates some limitations for understanding the natural history of ecDNA development. In addition, some primary cell lines are inherently challenging to establish and manipulate in culture – a feature that is explored in relation to imaging tools below. Finally, the examples above highlight the importance of high-quality clinical data for patient samples, particularly where patients have previously received treatment, and for this to remain clearly described in subsequent experimental work.

## Visualising ecDNA

In this section, we explore the options for visualising ecDNA using light microscopy, and how the data generated might be used to explore ecDNA function and organisation.

### Cytogenetic tools – DNA fluorescence in situ hybridisation (FISH)

**Fig. 2:**
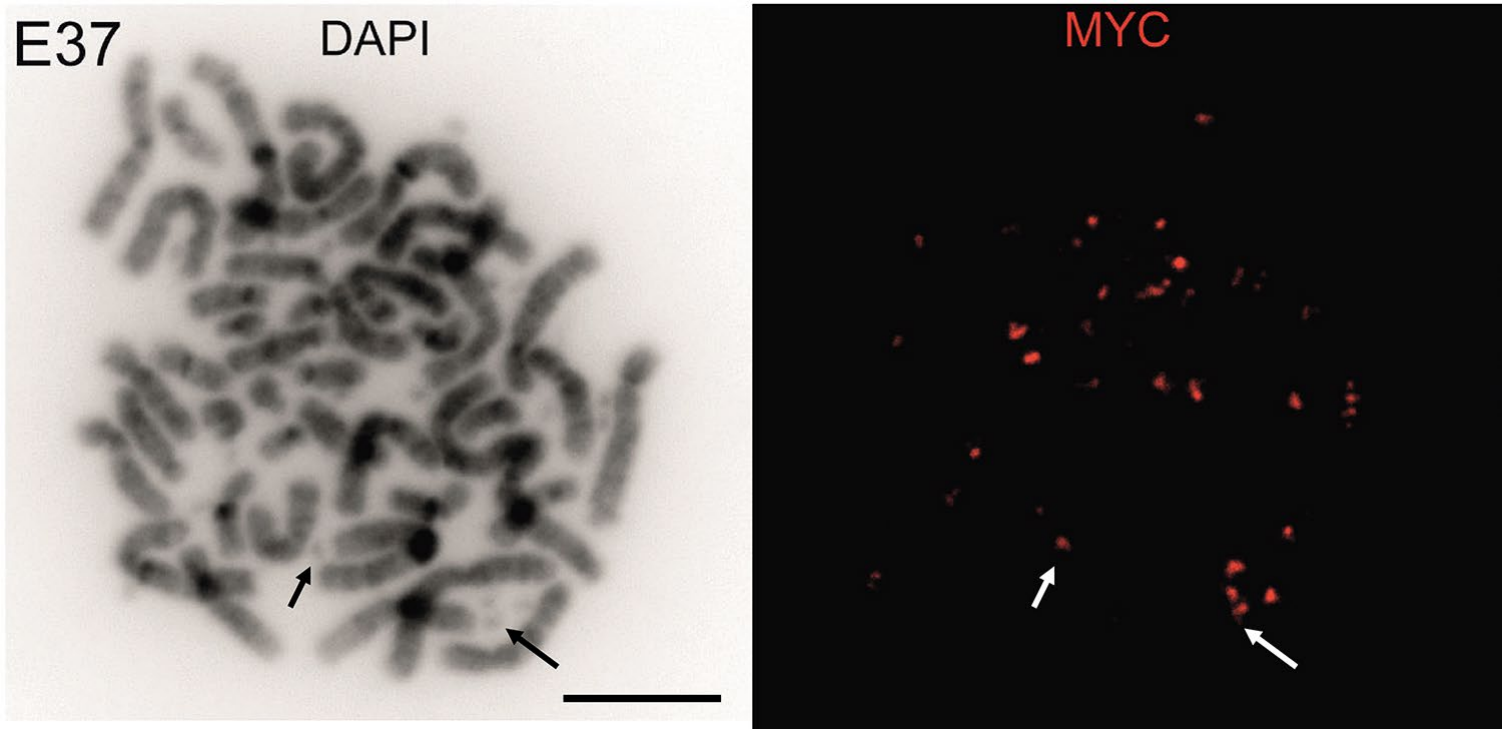
Detection of oncogenes on ecDNA by DNA-FISH. Left: DAPI stained metaphase spreads from a recurrent glioblastoma cell line E37. Right: DNA-FISH with a probe (red) detecting *c*-*MYC*. Arrows indicate examples of DNA-FISH signal on ecDNA for orientation. Scale bar: 10 µm

EcDNA were first described using simple light microscopy. They can be directly visualised on metaphase spreads via 4′,6-diamidino-2-phenylindole (DAPI) staining (Fig. 1), and DNA fluorescence in situ hybridisation (DNA-FISH) allows visualisation of genomic loci, including oncogenes and regulatory elements that are carried on ecDNA (van der Hout et al. 1989; Shapiro et al. 1993) (Fig. 2). RNA- FISH, with probes targeting intronic regions, can detect nascent RNA transcripts on ecDNAs (Purshouse et al. 2022). These cytogenetic tools remain the most robust way of characterising individual ecDNA in cells, their spatial organisation and ecDNA/HSR dynamics. However, these are low throughput methods, and it can be difficult to generate metaphases in some cell lines – an essential validation step to confirm WGS-predicted ecDNA. For example, in one study metaphase spreads could only be obtained from 72/117 (61.5%) cancer cell lines (Turner et al. 2017), and we found that extensive optimisation with alternative mitotic arrest agents was required in primary glioblastoma cells (Purshouse et al. 2022). FISH requires some prior knowledge of the sequences present on ecDNA and relies on high quality epifluorescence, or increasingly confocal-based, microscopy, particularly when combined with quantitative analysis. RNA- and DNA-FISH signals can be compared to quantify transcriptional efficiency but are difficult to combine without degradation of either signal. We have previously used sequential imaging of nuclei to capture RNA- and DNA-FISH signals to overcome this challenge (Purshouse et al. 2022).

Direct imaging remains the only method by which ecDNA and HSRs can be differentiated, with computational genomic tools unable to confidentially differentiate tandem repeats with current sequencing methods. Even then, this remains challenging owing to the varying definitions of an HSR, with some hybridisation signals appearing as large doublet foci on chromosomes (Shoshani et al. 2021) and others coating an entire chromosome arm (Storlazzi et al. 2010). Further tools are needed to differentiate ecDNA from HSRs, particularly as both of these are dynamic entities, including in response to selection pressures and DNA damage (Coquelle et al. 2002; Nathanson et al. 2014; Shoshani et al. 2021). Until such tools exist, the data presented here highlight the importance of combining sequencing tools with direct visualisation of genomic loci by DNA-FISH of metaphase spreads to accurately characterise ecDNA and HSRs.

EcDNA can also be visualised in nuclei by DNA-FISH to assess spatial organisation. This is particularly powerful in cells harbouring two or more ecDNA species, as this can resolve the issue of optic resolution being insufficient to discriminate between two closely located loci of the same species.

### Live-cell imaging

Unlike DNA-FISH which requires cell fixation, live-cell imaging would enable ecDNA dynamics to be monitored in real-time. Strategies for this are in development, but single-copy locus detection in live cells remains technically challenging. The Casilio system uses a dead-Cas9, guide RNAs (gRNAs) targeting ecDNA breakpoints and an RNA-aptamer to recruit multiple fluorescent reporter molecules to a single locus (Yi et al. 2021; Clow et al. 2022). This approach generated large foci of fluorescent RNA binding protein of varying sizes which look significantly larger and more irregular than the signals generated by FISH, raising some concern about aggregation of fluorescent molecules (Clow et al. 2022). The large foci of signal from probes targeted to ecDNA have been suggested to result from hubs of clustered ecDNA, with dual-colour ecDNA labelling of ecEGFR breakpoints used to subjectively call instances of colocalisation (Yi et al. 2021). However, the differences between signals generated by live cell imaging and by DNA-FISH need to be resolved.

Other systems, relying on the recruitment of fluorescent fusion proteins to integrated bacterial sequences, could be adapted for live cell imaging of ecDNA but would require the engineering of bacterial sequences into ecDNA (Germier et al. 2018; Alexander et al. 2019), something that has not yet been attempted. More recently, Cre-inducible ecDNA have been generated which express fluorescent proteins and that can be used to monitor some aspects of ecDNA biology (Pradella et al. 2023).

Live-cell imaging tools hold great potential for studying ecDNA dynamics but require further validation to mitigate concerns about fluorophore binding artefacts, as well as addressing uncertainties around binding affinity and effects on function.

### Combining DNA and protein imaging

**Fig. 3:**
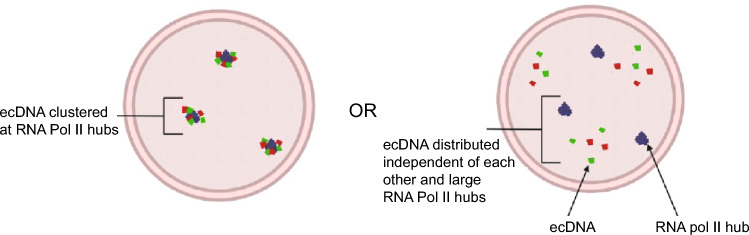
Left: hypothesis for ecDNA transcription driven by ecDNA–ecDNA and ecDNA–RNA Pol II hubs. Right: hypothesis that ecDNA (red and green) localise in the nucleus independent of each other and RNA Pol II hubs

Labelling of genomic loci combined with immunofluorescence enables the evaluation of locus localisation relative to nuclear proteins, such as RNA polymerase II (RNA Pol II). This is of interest owing to the condensate or ‘hubs’ hypothesis whereby key transcription factors, co-activators and RNA Pol II are suggested to concentrate together and partition away from the general nucleoplasm (Palacio and Taatjes 2022). However, given the small size of condensates, and their dynamic nature, investigating this hypothesis requires super-resolution, or preferably single molecule, imaging. The role of such regulatory hubs in driving gene expression remains a topic of active discussion (McSwiggen et al. 2019; Mir et al. 2019). Since ecDNA have large regions of accessible chromatin and harbour both oncogenes and their cognate enhancers, it has been proposed that ecDNA may cluster in hubs, enhancing their transcriptional output (Morton et al. 2019; Wu et al. 2019; Hung et al. 2021; Yi et al. 2021; Zhu et al. 2021) (Fig. 3). Whilst some imaging approaches have proposed greater-than-expected overlap between ecDNA and RNA Pol II signals (Yi et al. 2021), we did not identify a close spatial relationship between large RNA Pol II foci (> 500 nm) and ecDNA in glioblastoma cells (Purshouse et al. 2022).

## Image analysis strategies

### Qualitative analysis

Qualitative image analysis can characterise some features of ecDNA in metaphase spreads or interphase nuclei. Some inherent challenges are presented by this approach. Further tools are needed to differentiate ecDNA from HSRs on metaphase spreads beyond a qualitative description. There may be variations in FISH signal intensity in nuclei owing to probe accessibility and differences between cell lines that might influence conclusions drawn from a purely qualitative assessment of imaging data.

### Quantitative analysis

Quantitative analysis of microscopy images provides more unbiased insights into genome dynamics. These tools have progressed from simple methods to quantify the number of ecDNA in cell lines to those that explore the spatial organisation of ecDNA.

#### Two dimensional (2D) analysis

EcDetect (Turner et al. 2017), and later EcSeg (Rajkumar et al. 2019), were devised as automated tools for ecDNA counting using the DAPI signal, with the latter incorporating DNA-FISH to determine oncogene location on ecDNA or chromosomes. We have also used open-source imaging software, such as ImageJ, to automate counting of ecDNA (data not published). This can be useful in establishing fundamental features, such as ecDNA copy number range within and between cell lines, and how this is affected by cell passaging (Turner et al. 2017).

#### Three dimensional (3D) analysis

Diverging evidence exists as to whether ecDNA cluster together in the nucleus to form transcriptional hubs, with differing imaging and analysis approaches, definitions and models all likely contributing to these differences. Robustly determining the spatial organisation of ecDNA in the nucleus, relative to each other and to other nuclear landmarks, requires quantitative analysis of 3D images.

A study of established and primary cell lines used DNA-FISH and confocal microscopy to image ecDNA in nuclei with z stacks in 0.6 µm steps across approximately 8 µm (Hung et al. 2021). This large step size in z is too low to draw conclusions about localisation in transcriptional hubs – generally considered to be only a few hundred nanometres in size. The approach to clustering analysis of DNA-FISH signals was to use an autocorrelation function which assigned a random distribution as a control, with output defined in pixels. There was no control for ecDNA copy number. This analysis was performed on established cell lines, including Colo320DM. However, DNA-FISH for *c*-*MYC* on metaphase spreads shows that ecDNA in Colo320DM nuclei are large doublets approximately 1–2 μm in size each harbouring multiple copies of *c*-*MYC* per ecDNA. In support of this, Colo320DM ecDNA have previously been reconstructed at 4.33 Mb in size, measuring approximately 1.75 μm in diameter via imaging and harbouring three copies of *c*-*MYC* following detailed multimodal reconstruction (Wu et al. 2019; Hung et al. 2021). As such, some clustering of *c*-*MYC* hybridisation signals is inevitable owing to their structural co-localization on the same DNA molecule and it is hard to know how to control for this. Primary cell lines are also provided as evidence of clustering, however in the absence of metaphase spreads to verify these as ecDNA rather than HSRs, this limits further conclusions.

While not a quantitative analysis of the raw imaging data, another study proposing ecDNA clustering used live-cell imaging in a primary glioblastoma neurosphere culture, tagging EGFR-ecDNA breakpoints using the Casilio system (Yi et al. 2021).

**Fig. 4:**
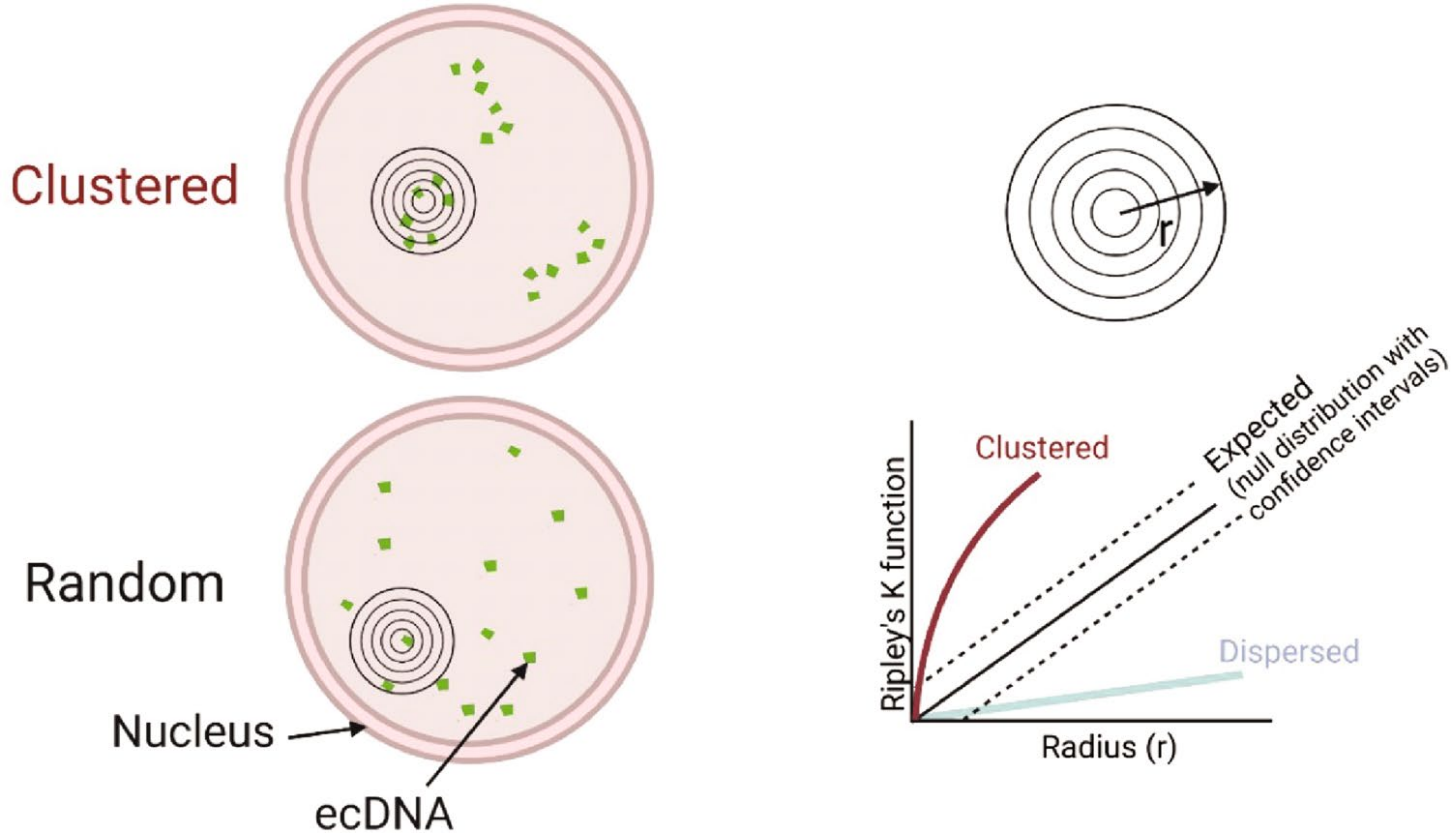
Schematic of nuclei showing clustered (top) and random (bottom) distribution of DNA-FISH signals detecting ecDNA, with overlay of increasing radii (*r*) to indicate Ripley’s K. The graph shows how this is plotted per nucleus, with the expected values ± confidence interval shown to represent the null distribution, i.e. random distribution. Observed values within this null distribution would be considered randomly distributed. The lines indicate observed values as they would plot if foci were clustered (burgundy) or dispersed (blue). Adapted from Purshouse et al. 2022

We sought to address the challenges of determining whether there is clustering of ecDNA in the nucleus by developing a method using Ripley’s K function (Fig. 4). Ripley’s K analysis allows for ecDNA copy number and nuclear size to be controlled for in each individual nucleus. We compared observed and expected point patterns of DNA-FISH signals using spinning disc confocal microscopy to image nuclei of glioblastoma cells in 0.1 µm z steps across 3 µm (Purshouse et al. 2022), with the intention of focussing on ecDNA–ecDNA distances that might be associated with coordinated transcription in hubs (< 200 nm). We did not observe ecDNA clustering at such distances (Purshouse et al. 2022). Our data was suggestive of the spatial freedom of ecDNA relative to chromosomes and a regional localisation more reflective of the non-random organisation of the nucleus into chromosome territories and A/B compartments.

## An overview of bioinformatic tools for analysing ecDNA

While the focus of this review is to explore the role of imaging in investigating ecDNA, DNA sequencing-based analysis tools have markedly advanced our understanding of ecDNA biology. We highlight how these tools can complement imaging data.

### AmpliconArchitect

AmpliconArchitect (AA) is a tool for ecDNA amplicon reconstruction from paired-end WGS data (Turner et al. 2017; Deshpande et al. 2019). It has been developed further to the AmpliconSuite analysis pipeline (Luebeck et al. 2023), which incorporates AmpliconClassifer (for output classification) (Kim et al. 2020) and CNVkit for calling copy number variation (CNV) and alterations across the genome (Talevich et al. 2016). AA uses WGS data to link CNV regions of increased copy number and identifies linked segments. This builds on Circle-seq, a rolling circle amplification tool that preferentially identifies shorter circular DNA (e.g. eccDNA) (Møller et al. 2018; Kim et al. 2020).

However, short read WGS data may be unable to span the long repeats on ecDNA, limiting the ability to differentiate between multiple possible structures. This in turn can result in multiple possible candidate amplicons being output, from amongst which the user must then choose their ‘amplicon of interest’, possibly resulting in reporting bias (Deshpande et al. 2019). In addition, correlation of AA with DNA-FISH on metaphase spreads suggested an 85% positive predictive value of amplicons characterised as ‘circular’ by AA as corresponding to extrachromosomal DNA-FISH signal. The sensitivity of AA to identify circular ecDNA was 83% (i.e. 83% of signals identified via DNA-FISH as being extrachromosomal were also classified as ‘circular’ by AA). AA is also unable to differentiate between ecDNA and HSRs and may miscall shape (e.g. circular versus linear). As such, direct visualisation by DNA-FISH remains the ‘gold standard’ modality for verifying whether a multicopy amplification identified via WGS and AA is either an ecDNA or an HSR.

The gastric cancer cell line SNU16, which harbours *FGFR2* and *c*-*MYC* oncogenes on ecDNA, was analysed via a novel ecDNA isolation and analysis methodology, CRISPR-CATCH. This identified many subspecies of ecDNA (Hung et al. 2022), including *c*-*MYC* and *FGFR2* ecDNA hybrids, which were validated by DNA-FISH.

### AmpliconReconstructor

EcDNA may be more accurately characterised by combining long-read sequencing and optical mapping, and analysis with AmpliconReconstructor (Wu et al. 2019; Luebeck et al. 2020; Hung et al. 2022). Long reads are more likely to span across breakpoints, provide detailed structural variant data and be able to report tandem repeats, such as those seen in HSRs. Long read sequencing has been combined with novel in vitro techniques to enrich for ecDNA and to characterise ecDNA heterogeneity at ever-higher resolution. For example, ecDNA in cancer cells were digested and the DNA amplified using exonucleases, followed by ecDNA characterisation using Circle-seq (Koche et al., 2020). CRISPR-CATCH represents a more targeted approach to characterise ecDNA, although this requires an underlying knowledge of likely ecDNA sequences for CRISPR-targeted guide design (Jiang et al. 2015; Hung et al. 2022).

### Single nucleotide polymorphism (SNP) analysis

To address the limitations of multi-copy oncogene analysis from sequencing data we, and others, have utilised single nucleotide polymorphism (SNP) analysis to evaluate ecDNA transcriptional efficiency. Genomic events that give rise to ecDNA originate from only one of the parental copies of a chromosome (Stephens et al. 2011; Hung et al. 2022). We used SNPs present in the exons of oncogenes amplified on ecDNAs, in cell lines heterozygous for these SNPs, to determine the ratio of the SNP alleles in transcripts (RNA-sequencing data) and in the genome (WGS). This demonstrated that the transcriptional efficiency of *EGFR* was comparable between ecDNA and chromosomal *EGFR* loci (Purshouse et al. 2022). A similarly linear relationship was also observed in a study of ecDNA copy number and gene expression in primary neuroblastoma (Stöber et al. 2023).

## Chromatin accessibility and nuclear organisation

EcDNA chromatin organisation has been explored using various genomic tools. Chromosome conformation capture assays have captured ecDNAs associated with transcriptionally active chromosomal regions in two ecDNA-harbouring GBM-patient derived cell lines (Zhu et al. 2021). In a pan-cancer study, ecDNA were shown to have large regions of accessible chromatin (assayed by ATAC-seq), indicative of nucleosome displacement by bound transcription factors, and to be decorated with histone modifications associated with active chromatin (Wu et al. 2019). These are both features typically associated with active regions in the centre of the nucleus, away from the nuclear periphery (Bickmore 2013). In support of this, imaging analysis from our laboratory, and others, have described a preferential distribution of ecDNA towards the centre of the nucleus (Lundberg et al. 2008; Purshouse et al. 2022). In contrast, early studies in Colo320 cells reported that *ec*-*MYC* ecDNA (and HSRs) were preferentially localised at the nuclear periphery, a region typically associated with heterochromatin and transcriptional repression, moving more internally during S-phase (Itoh and Shimizu 1998). These diverging observations highlight ongoing uncertainty about ecDNA nuclear dynamics and the importance of working with diverse cellular models.

## Single-cell extrachromosomal circular DNA and transcriptome sequencing (scEC&T-seq)

Novel bioinformatic tools increasingly seek to tease apart the inter- and intra-cellular heterogeneity of ecDNA. ScEC&T-seq allows for RNA and DNA sequencing from the same single cells; recent studies have used this approach in primary neuroblastoma cells and cell lines to describe not only the overall linear relationship between ecDNA copy number and gene expression, but also to propose highly heterogeneous gene expression and transcriptional states within individual neuroblastoma patients (Stöber et al. 2023; Chamorro González et al. 2023).

Although ‘omic’ approaches give invaluable information on ecDNA sequence and some aspects of ecDNA structure and chromatin organisation, these approaches are unable to determine some key features of ecDNA behaviour, such as their spatial organisation and dynamics within the nucleus. Imaging remains the key modality for addressing these questions. While some studies have previously combined imaging with sequencing to characterise bulk cell populations, the future likely lies in true combinatorial strategies. A recent study combining FISH and genomic data at single cell resolution in a glioblastoma cohort (Walentynowicz et al. 2023) offers some insight into the potential of spatial transcriptomics in exploring spatial, as well as temporal, ecDNA dynamics.

## Conclusions

EcDNA clearly represent a major mechanism through which cancer cells can amplify oncogenes. However, it remains unclear whether ecDNA represent an important targetable entity. Despite being described for the first time almost 60 years ago by simple light microscopy, novel scientific tools are enabling the study of ecDNA in greater detail. While computational analysis of sequencing data offers significant opportunities to understand ecDNA dynamics, imaging can offer unique insights into the 3D organisation of ecDNA that sequencing cannot address. Coupling developments in advanced imaging, in 3D and in live cells, with sequence-based analyses promises to provide much needed understanding of how ecDNA contribute to cancer progression and response to treatments. Whilst cell line, organoid and xenograft model systems will provide the platforms to support much of this work, the analysis of ecDNA in primary tumour material will also be important, particularly for the understanding of ecDNA in the context of intra-tumoural heterogeneity.
